# Socioeconomic and ethnic inequalities in oral health among children and adolescents living in England, Wales and Northern Ireland

**DOI:** 10.1111/cdoe.12390

**Published:** 2018-06-10

**Authors:** Patrick Rouxel, Tarani Chandola

**Affiliations:** ^1^ CLOSER Department of Social Science University College London Institute of Education London UK; ^2^ UCL Eastman Dental Institute London UK; ^3^ Social Statistics University of Manchester Manchester UK

**Keywords:** disparities, ethnicity, oral health, public health, socioeconomic inequalities

## Abstract

**Objectives:**

Although adolescence is a sensitive developmental period in oral health, the social equalization hypothesis that suggests health inequalities attenuate in adolescence has not been examined. This study analyses whether the socioeconomic gap and ethnic disadvantage in oral health among children aged 5 reduces among adolescents aged 15.

**Methods:**

Data from the cross‐sectional Children's Dental Health Survey 2013 were analysed, comprising of 8541 children aged 5, 8, 12 and 15 attending schools in England, Wales and Northern Ireland. Oral health indicators included decayed and filled teeth, plaque, gingivitis and periodontal health. Ethnicity was measured using the 2011 UK census ethnic categories. Socioeconomic position was measured by family, school and residential deprivation. Negative binomial and probit regression models estimated the levels of oral health by ethnicity and socioeconomic position, adjusted for demographic and tooth characteristics.

**Results:**

The predicted rate of decayed teeth for White British/Irish children aged 5 was 1.54 (95%CI 1.30‐1.77). In contrast, the predicted rate for Indian and Pakistani children was about 2‐2.5 times higher. At age 15, ethnic differences had reduced considerably. Family deprivation was associated with higher levels of tooth decay among younger children but not among adolescents aged 15. The influence of residential deprivation on the rate of tooth decay and filled teeth was similar among younger and older children. Moreover, inequalities in poor periodontal health by residential deprivation was significantly greater among 15‐year‐old children compared to younger children.

**Conclusions:**

This study found some evidence of smaller ethnic and family socioeconomic differences in oral health among British adolescents compared to younger children. However, substantial differences in oral health by residential deprivation remain among adolescents. Community levels of deprivation may be particularly important for the health of adolescents.

## 1 INTRODUCTION

There are substantial social inequalities in oral health, with children from disadvantaged and ethnic minority backgrounds experiencing poorer oral health.^1^, ^2^ Furthermore, there is considerable evidence that socioeconomic health inequalities appear to reduce from childhood to adolescence leading to the hypothesis of social equalization during adolescence.^3^, ^4^, ^5^, ^6^ This hypothesis involves a change in the pattern of socioeconomic differences in health from 1 in childhood characterized by health inequalities to 1 in youth characterized by relative equality.^3^ However, the social equalization hypothesis in oral health has not been explicitly examined in previous studies.

Despite being preventable, dental caries and periodontal diseases are the 2 most prevalent oral diseases contributing to the global burden of chronic diseases.^7^, ^8^ While the prevalence of caries among children and adolescents living in the UK has reduced considerably over the last 40 years, caries is now concentrated in a minority of the child population.^9^ Children from disadvantaged family socioeconomic backgrounds have a higher risk of dental caries^1^ and periodontal disease.^10^

Oral health inequalities by ethnicity^11^ and area deprivation^12^ have also been reported. In the UK, there is a high level of caries experience among preschool and school children from Pakistani, Bangladeshi, Chinese and East European backgrounds, even after controlling for levels of socioeconomic deprivation.^2^, ^13^, ^14^ Afro‐Caribbean children generally had better or similar oral health than White children.^2^ Socioeconomic position (SEP) and ethnicity appear to influence caries or periodontal disease by influencing oral health behaviours,^15^ in particular, oral hygiene habits, diet, smoking and dental attendance.^16^, ^17^

Reductions in socioeconomic inequalities as children age are apparent for several major single health conditions such as accidents, injuries and mental health^3^ and for longstanding illnesses, psychosocial well‐being and obesity.^6^ However, similar reductions in ethnic differences in health as children grow older have not been reported, even though ethnic minority children tend to live in poorer socioeconomic circumstances. There is also some evidence that socioeconomic inequalities in oral health are attenuated among older children compared to younger children.^18^, ^19^ This socioeconomic equalization in health during adolescence is suggested to arise when the influences of the family and home environment diminish, with school, peers and youth culture playing a larger role in children's lives.^3^ Adolescence is a period when oral health‐related behaviours are not as closely monitored by parents as during childhood, with a potentially larger role for the school and neighbourhood factors in influencing adolescent oral health and related behaviours. In addition, the transition from childhood to adolescence is a sensitive developmental period in oral health with the replacement of primary teeth by permanent teeth. A reduction in tooth decay in early adolescence may reflect the lower lifetime exposure of permanent teeth to oral health risk factors.

This study examines whether the association between ethnicity, SEP and oral health differs in childhood and mid‐adolescence. We hypothesize that family SEP and ethnicity will be less influential for the oral health of adolescents compared to younger children living in England, Wales and Northern Ireland.

## 2 METHODS

### 2.1 Data source

The Children's Dental Health Survey (CDHS) 2013 was based on a representative sample of children aged 5, 8, 12 and 15 attending state‐funded and independent schools across England, Wales and Northern Ireland using a multistage cluster random sampling design with oversampling of disadvantaged children and children in Wales and Northern Ireland. Response within each age group also differed between the countries. From the 13 628 target sample, 9866 children completed a dental examination. The examinations were performed in schools by trained and calibrated examiners using standardized measurement protocols. Full details of the sample design and methodology can be found elsewhere.^20^ In order to make the sample estimates representative of children living in England, Wales and Northern Ireland, the survey team produced a dental examination weight that took account of the survey design, adjusted for nonresponse, which was calibrated to population totals. Families living in poor areas, children aged 8, girls and White children were less likely to respond. The survey received ethical approval from the Research Ethics Committee at University College London. (UCL Research Ethics Committee, Project ID: 2000/003).

### 2.2 Variables

We selected multiple clinical indicators to capture the different aspects of children's oral health that include the condition of their teeth and gums (further details in Supporting Information). At all ages, the analyses included both the primary and the permanent teeth because of the mixed dentition of the children. At age 5 and 8, 47% and 99% of the children had at least 1 permanent tooth erupted, out of which 4% at age 5 and 38% at age 8 had experienced caries.

Clinical oral health outcomes included the number of decayed teeth. In line with developments in epidemiological studies, the CDHS 2013 adopted the International Caries Detection and Assessment System for assessing first staging of caries process.^21^ We therefore used the number of teeth affected at the clinical decay threshold, which includes untreated obvious decay (visual and cavitated decay into dentine) and visual and cavitated decay into enamel. The number of filled teeth was used as an indicator of access to dental services and receipt of dental care. We also analysed an indicator of poor periodontal health by combining the presence of some gum inflammation (gingivitis), the presence of plaque or the presence of calculus in more than 1 sextant. Separate analyses of gingivitis and plaque are presented in Tables S7 and S8. Gingivitis is a reversible periodontal condition that is prevalent among children and adolescents^10^ and is particularly associated with puberty or presence of mixed dentition.^22^ Dental plaque is the main biological aetiological factor for the development of dental caries and periodontal diseases.^23^

Missing teeth were not analysed as a separate dependent variable, as the reason for missing teeth in the primary dentition was not recorded. Missing primary teeth could be due to decay or natural exfoliation, and hence, to allow comparison for all age groups, the decayed, missing and filled teeth (dmft/DMFT) index was not used to measure caries experience. In the permanent dentition, there were very few children with missing teeth due to decay (4.8%).

Ethnicity of the children was collected from school records, which used parents' reporting of family ethnic group when their child started at school, and was assessed using the 2011 UK census ethnic categories.^24^ The 8 ethnic groups used for analysis were: White British/Irish; other White background; Mixed White (White & Black Caribbean/African/Asian); Indian; Pakistani; Bangladeshi; Black African; and Black Caribbean/other Black African‐Caribbean. The other White children in the CDHS largely represent children of Eastern European origin, as the survey over sampled schools in deprived areas where Eastern European origin families are more likely to live.^25^ We excluded children from Chinese, other Asian, Arab, Gypsy and Irish travellers and “Other” unspecified ethnic groups due to small numbers or the heterogeneous nature of the “Other” category.

Socioeconomic variables included free school meals eligibility (at the child level): a statutory benefit available for children from families who received income related benefits and is a proxy for family level relative income deprivation;^20^ deprived school: schools with more than 30% of children eligible for free school meals were defined as “deprived”; and the Index of Multiple Deprivation (IMD) as a marker of small area residential deprivation. The latest index for each country was used at the time of linking, which was the 2010 Index for England; the 2011 index for Wales; and the 2010 Northern Ireland Multiple Deprivation Measure. The IMD quintiles were transformed into weighted rank scores, ranging from children living in the most deprived areas to those from the least deprived areas. These rank scores were calculated relative to each country, with the weights accounting for the proportional distribution of the children within each IMD quintile (and country).

Covariates included in the regression models were the child's sex, urban/rural residence and country. As the pattern of caries is age dependent, and the tooth eruption age distributions result in caries pattern differences that vary by age, the models also controlled for the number of primary and permanent teeth.

### 2.3 Statistical analysis

Statistical analyses were undertaken in STATA using a design factor to take account of the complex sampling and weighting procedures. The weights derived by the CDHS survey team explicitly takes into account the pattern of missingness by ethnicity and SEP.^20^

From the sample of 9866 children who completed a dental examination, we excluded 1090 children due to item nonresponse on explanatory variables. The overall rate of missingness was 11.1%, of which ethnicity accounted for 5.7%; free school meals 4.1% and IMD 4.2%. We also excluded children from “Other” ethnic groups (n = 235). Therefore, the sample was reduced to 8541 children: 5‐year‐olds n = 2217 (26.4%); 8‐year‐olds n = 2083 (24.3%); 12‐year‐olds n = 2183 (25.5%) and 15‐year‐olds n = 2058 (24.8%).

The relative index of inequality (RII) was used to summarize the magnitude of inequalities in oral health between the IMD quintiles. The RII is especially useful in this analysis because it allows us to compare the IMD inequalities across different children's ages, even though the rate of poor oral health differs by age. The RII was generated from the country‐specific IMD quintiles using the subgroup option of the STATA program file “riigen.”^26^ We additionally tested for country differences in the association of RII with the different oral health outcomes by including an interaction term between the RII and country in the regression models predicting oral health (Table S9).

It is important to ensure, empirically, that the effects of family SEP on children oral health are estimated net of individual ethnic minority status. Previous research shows that ethnic minority status influences health independently of income, education and socioeconomic characteristics.^27^ There was no indication of colinearity between ethnicity and the different SEP indicators, as the regression coefficients and standard errors were relatively stable with different models.

We used negative binomial regression to model the count variables (clinical decay and filled teeth). As the number of teeth varies between children, these models estimated the rate of tooth decay and fillings (per child) by including the log number of teeth as an offset.

We used probit regression to model the binary outcome variables (gingivitis, plaque, poor periodontal health). We reported the predicted probabilities and rates of each of the outcomes by the ethnicity and SEP categories holding all the other variables in the models at their means. We examined the equalization hypothesis by testing whether the interaction between SEP/ethnicity and age group was significant. The reference group for the measures of SEP and ethnicity was always the most advantaged group in all the analyses—White British and Irish ethnicity, not eligible for free school meals, not attending a deprived school and least deprived residential area.

We conducted a series of sensitivity analyses to assess the robustness of our findings (Tables S5‐S10). Methodological differences and lack of data on ethnicity and socioeconomic positions in the previous CDHS surveys prevented trend analyses. For dental decay, we repeated the negative binomial regression analyses using the 2003 criteria (Table S6). We also conducted multilevel logistic analyses, analysing the presence of tooth decay at the tooth level (level 1) clustered within children (level 2) (Table S10).

## 3 RESULTS

The distribution of all the variables by the 4 age‐cohort samples is displayed in Table 1. For reference, the mean number of decayed, filled, primary and permanent teeth, as well as the percentage of children with gingivitis, plaque and poor periodontal health for each age‐cohort are presented in Tables S1‐S4.

**Table 1:** Distribution of children by demographic and socioeconomic characteristics in the 4 age‐cohort samples (Children Dental Health Survey 2013): unweighted N/weighted %

Sample characteristics	N = 2217 (26.4%)	N = 2083 (24.3%)	N = 2183 (24.5%)	N = 2058 (24.8%)
	5 y	8 y	12 y	15 y
Child's gender				
Boys	1082 (50.5)	1033 (51.4)	1061 (51.6)	992 (48.7)
Girls	1135 (49.5)	1050 (48.6)	1122 (48.4)	1066 (51.3)
Output area classification				
Urban	1759 (88.2)	1626 (87.9)	1862 (88.6)	1733 (87.9)
Rural	458 (11.9)	457 (12.1)	321 (11.4)	325 (12.1)
Country				
England	1279 (91.2)	1163 (90.7)	1220 (91.0)	1123 (90.5)
Wales	425 (4.9)	429 (5.4)	539 (5.5)	464 (5.8)
Northern Ireland	513 (3.9)	491 (3.9)	424 (3.5)	471 (3.8)
Ethnicity				
White British & Irish	1832 (76.6)	1752 (78.6)	1799 (78.5)	1698 (79.7)
Other White	105 (7.0)	86 (6.2)	56 (3.6)	55 (3.5)
Mixed White	85 (4.9)	56 (3.8)	55 (3.3)	54 (4.2)
Indian	27 (1.9)	27 (1.3)	40 (2.1)	48 (2.4)
Pakistani	56 (2.5)	52 (2.3)	77 (3.7)	72 (2.7)
Bangladeshi	29 (1.4)	26 (1.9)	64 (3.4)	55 (3.4)
Black African	38 (2.2)	46 (3.5)	50 (3.2)	37 (2.3)
Black Caribbean	45 (3.5)	38 (2.4)	42 (2.4)	39 (1.9)
Free school meal				
Not eligible	1711 (81.9)	1650 (84.2)	1591 (78.8)	1579 (82.8)
Eligible	506 (18.1)	433 (15.8)	592 (21.2)	479 (17.2)
Deprived school				
No	1510 (79.1)	1405 (78.9)	1348 (77.4)	1215 (77.4)
Yes	707 (20.9)	678 (21.1)	835 (22.6)	843 (22.6)
Index of Multiple Deprivation quintiles (home postcode)				
England				
Least deprived	151 (16.5)	143 (16.7)	151 (15.5)	143 (15.8)
Quintile 2	170 (17.0)	157 (17.4)	172 (18.7)	153 (18.1)
Quintile 3	221 (21.6)	176 (19.6)	141 (11.6)	135 (15.1)
Quintile 4	239 (16.4)	252 (19.0)	225 (19.1)	200 (17.6)
Most deprived	498 (28.5)	435 (27.3)	531 (35.2)	492 (33.4)
Wales				
Least deprived	37 (18.0)	36 (19.7)	42 (9.6)	36 (9.0)
Quintile 2	88 (22.7)	94 (21.0)	87 (17.0)	80 (21.1)
Quintile 3	76 (21.2)	94 (21.2)	91 (16.6)	75 (17.0)
Quintile 4	93 (19.3)	94 (19.9)	142 (31.4)	128 (32.9)
Most deprived	131 (18.7)	111 (18.3)	177 (25.4)	145 (20.0)
Northern Ireland				
Least deprived	61 (10.8)	59 (13.6)	36 (13.8)	40 (11.1)
Quintile 2	119 (25.0)	99 (18.7)	68 (20.0)	68 (16.7)
Quintile 3	166 (28.7)	165 (29.1)	105 (23.3)	104 (20.5)
Quintile 4	119 (19.8)	119 (22.0)	96 (22.4)	125 (28.2)
Most deprived	48 (15.7)	49 (16.7)	119 (20.5)	134 (23.5)

Tables 2, 3 and 4 present the results of the regression models with the different oral health outcomes at age 5, 8, 12 and 15. Only the predicted probabilities by ethnicity and SEP are shown, although all the models control for sex, urban/rural, country and the number of primary and permanent teeth.

**Table 2:** Predicted Rates (PRs) and 95% confidence intervals (CIs) from negative binomial regression models^a^,^b^ clinical tooth decay in children regressed on ethnicity and socioeconomic position in the 4 age‐cohort samples (Children Dental Health Survey 2013)

Variables	N = 2217	N = 2083	N = 2183	N = 2058	Age interaction Ethnicity/SEP
	5 y	8 y	12 y	15 y	
	PR (95% CI)	PR (95% CI)	PR (95% CI)	PR (95% CI)	
Ethnicity					
White British &Irish	1.54 (1.30, 1.77)	2.24 (1.92, 2.55)	1.76 (1.50, 2.02)	1.88 (1.40, 2.36)	
Other White	2.04 (1.39, 2.69)	**3.27 (2.20, 4.33)** *	2.01 (1.01, 3.00)	1.44 (0.46, 2.42)	
Mixed White	1.79 (1.03, 2.54)	2.37 (1.32, 3.42)	1.30 (0.67, 1.93)	2.16 (1.07, 3.25)	
Indian	**3.71 (1.08, 6.34)** *	3.32 (1.35, 5.29)	1.28 (0.46, 2.11)	1.14 (0.45, 1.82)	
Pakistani	**2.85 (1.85, 3.85)** **	**3.29 (2.36, 4.22)** *	1.90 (1.20, 2.60)	1.71 (0.59, 2.82)	
Bangladeshi	2.43 (0.76, 4.11)	2.54 (1.10, 3.99)	1.08 (0.35, 1.82)	1.40 (0.60, 2.20)	
Black African	**0.56 (0.26, 0.87)** **	1.28 (0.53, 2.03)	**0.69 (0.41, 0.97)** ***	1.65 (0.65, 2.64)	
Black Caribbean	1.21 (0.41, 2.01)	1.76 (1.19, 2.33)	1.30 (0.58, 2.02)	1.51 (0.58, 2.44)	
*F* test *P* value (*df*)	**<.001 (7)**	**.02 (7)**	**.001 (7)**	.30 (7)	**<.001 (21)**
Free school meal					
Not eligible	1.48 (1.26, 1.69)	2.27 (1.99, 2.54)	1.51 (1.28, 1.73)	1.79 (1.37, 2.21)	
Eligible	**2.22 (1.75, 2.68)** ***	2.53 (2.07, 2.99)	**2.25 (1.83, 2.68)** ***	1.94 (1.45, 2.45)	
*F* test *P* value (*df*)	**.003 (1)**	.19 (1)	**<.001 (1)**	.46 (1)	**.03 (3)**
Deprived school					
No	1.55 (1.31, 1.79)	2.31 (1.99, 2.63)	1.79 (1.51, 2.07)	1.78 (1.34, 2.22)	
Yes	1.82 (1.41, 2.24)	2.32 (2.00, 2.64)	1.34 (0.91, 1.76)	1.93 (1.18, 2.69)	
*F* test *P* value (*df*)	.21 (1)	.95 (1)	.13 (1)	.71 (1)	**.01 (3)**
IMD rank					
Least deprived	1.11 (0.83, 1.40)	1.77 (1.34, 2.20)	1.03 (0.72, 1.33)	1.43 (0.76, 2.10)	
Most deprived	**2.55 (1.91, 3.19)** ***	**3.28 (2.47, 4.09)** **	**2.92 (2.10, 3.75)** ***	2.44 (1.71, 3.16)	
*F* test *P* value (*df*)	**<.001 (1)**	**.006 (1)**	**<.001 (1)**	.12 (1)	.27 (3)

Bold text indicates a statistically significant difference with a *p*‐value less than .05.

^a^Survey weighted models include ethnicity, all socioeconomic variables, sex, country, urban/rural, and number of permanent and primary teeth.

^b^Negative binomial regression models include an offset (log number of teeth).

*P< .05; **P< .01; ***P< .001.

**Table 3:** Predicted Rates (PRs) and 95% confidence intervals (CIs) from negative binomial regression models^a^,^b^: filled teeth in children regressed on ethnicity and socioeconomic position in the 4 age‐cohort samples (Children Dental Health Survey 2013)

Variables	N = 2217	N = 2083	N = 2183	N = 2058	Age interaction Ethnicity/SEP
	5 y	8 y	12 y	15 y	
	PR (95% CI)	PR (95% CI)	PR (95% CI)	PR (95% CI)	
Ethnicity					
White British &Irish	0.10 (0.07, 0.13)	0.39 (0.28, 0.49)	0.39 (0.30, 0.48)	0.84 (0.71, 0.98)	
Other White	**0.30 (0.06, 0.53)** **	0.45 (0.25, 0.66)	0.61 (0.06, 1.16)	1.06 (0.66, 1.46)	
Mixed White	0.08 (0.02, 0.15)	0.36 (0.12, 0.59)	0.47 (0.09, 0.85)	1.25 (0.44, 2.06)	
Indian	0.22 (−0.07, 0.51)	0.60 (0.10, 1.10)	0.28 (0.11, 0.45)	0.68 (0.76, 0.99)	
Pakistani	0.14 (0.01, 0.26)	0.53 (0.18, 0.87)	0.56 (−0.02, 1.15)	0.55 (0.27, 0.84)	
Bangladeshi	0.13 (−0.01, 0.26)	0.27 (−0.10, 0.65)	0.29 (0.04, 0.53)	**0.33 (0.21, 0.44)** ***	
Black African	0.31 (−0.10, 0.71)	0.74 (0.32, 1.16)	0.29 (0.04, 0.54)	0.37 (−0.04, 0.77)	
Black Caribbean	**0.03 (0.01, 0.07)** *	**0.13 (0.01, 0.25)** *	0.37 (0.09, 0.64)	0.53 (0.12, 0.94)	
*F* test *P* value (*df*)	**.004 (7)**	**.002 (7)**	.64 (7)	**<.001 (7)**	**<.001 (21)**
Free school meal					
Not eligible	0.14 (0.09, 0.18)	0.40 (0.31, 0.50)	0.37 (0.30, 0.45)	0.79 (0.67, 0.91)	
Eligible	0.08 (0.04, 0.13)	0.40 (0.29, 0.50)	0.49 (0.29, 0.69)	0.94 (0.68, 1.20)	
*F* test *P* value (*df*)	.052 (1)	.92 (1)	.1 (1)	.27 (1)	.25 (3)
Deprived school					
No	0.12 (0.08, 0.17)	0.39 (0.29, 0.50)	0.43 (0.30, 0.57)	0.82 (0.67, 0.97)	
Yes	0.13 (0.07, 0.19)	0.42 (0.27, 0.57)	0.31 (0.18, 0.43)	0.80 (0.62, 0.97)	
*F* test *P* value (*df*)	.66 (1)	.79 (1)	.28 (1)	.82 (1)	.54 (3)
IMD rank					
Least deprived	0.08 (0.02, 0.14)	0.37 (0.23, 0.50)	0.37 (0.23, 0.51)	0.54 (0.37, 0.72)	
Most deprived	0.21 (0.09, 0.32)	0.45 (0.11. 0.79)	0.43 (0.27, 0.59)	**1.32 (0.88, 1.77)** **	
*F* test *P* value (*df*)	.09 (1)	.70 (1)	.65 (1)	**.006 (1)**	.53 (3)

Bold text indicates a statistically significant difference with a *p*‐value less than .05.

^a^Survey weighted models include ethnicity, all socioeconomic variables, sex, country, urban/rural, and number of permanent and primary teeth.

^b^Negative binomial regression models include an offset (log number of teeth).

*P < .05; **P < .01; ***P < .001.

**Table 4:** Predicted Probabilities (PPs) and 95% confidence intervals (CIs) from probit regression models^a^: poor periodontal health^b^ in children regressed on ethnicity and socioeconomic position in the 4 age‐cohort samples (Children Dental Health Survey 2013)

Variables	N = 2217	N = 2083	N = 2183	N = 2058	Age interaction Ethnicity/SEP
	5 y	8 y	12 y	15 y	
	PR (95% CI)	PR (95% CI)	PR (95% CI)	PR (95% CI)	
Ethnicity					
White British and Irish	0.45 (0.36, 0.54)	0.72 (0.66, 0.78)	0.75 (0.67, 0.83)	0.69 (0.59, 0.78)	
Other White	0.39 (0.24, 0.54)	0.68 (0.54, 0.82)	0.71 (0.56, 0.86)	0.70 (0.55, 0.85)	
Mixed White	0.58 (0.39, 0.77)	0.82 (0.70, 0.94)	0.73 (0.54, 0.92)	0.67 (0.47, 0.87)	
Indian	0.49 (0.27, 0.72)	0.73 (0.49, 0.96)	0.73 (0.58, 0.89)	0.54 (0.28, 0.81)	
Pakistani	0.56 (0.31, 0.82)	0.77 (0.59, 0.95)	0.74 (0.59, 0.88)	0.73 (0.55, 0.90)	
Bangladeshi	0.68 (0.45, 0.91)	0.74 (0.50, 0.98)	**0.88 (0.77, 0.98)** *	0.67 (0.50, 0.83)	
Black African	0.24 (0.04, 0.44)	0.68 (0.54, 0.82)	0.63 (0.44, 0.81)	0.80 (0.58, 1.02)	
Black Caribbean	**0.24 (0.11, 0.37)** **	0.59 (0.42, 0.76)	0.74 (0.56, 0.93)	0.64 (0.43, 0.85)	
*F* test *P* value (*df*)	**<.001 (7)**	.47 (7)	**.003 (7)**	.72 (7)	.18 (21)
Free school meal					
Not eligible	0.44 (0.35, 0.52)	0.72 (0.67, 0.77)	0.75 (0.67, 0.83)	0.68 (0.58, 0.78)	
Eligible	0.48 (0.36, 0.59)	0.69 (0.58, 0.80)	0.72 (0.63, 0.81)	0.71 (0.59, 0.83)	
*F* test *P* value (*df*)	.41 (1)	.43 (1)	.28 (1)	.58 (1)	.53 (3)
Deprived school					
No	0.44 (0.35, 0.53)	0.70 (0.64, 0.77)	0.73 (0.63, 0.83)	0.68 (0.57, 0.79)	
Yes	0.46 (0.36, 0.56)	0.77 (0.71, 0.82)	0.81 (0.76, 0.86)	0.71 (0.58, 0.84)	
*F* test *P* value (*df*)	.72 (1)	.11 (1)	.16 (1)	.67 (1)	.52 (3)
IMD rank					
Least deprived	0.45 (0.31, 0.58)	0.75 (0.67, 0.82)	0.68 (0.58, 0.77)	0.55 (0.39, 0.70)	
Most deprived	0.44 (0.31, 0.57)	0.67 (0.57, 0.77)	**0.83 (0.73, 0.93)** *	**0.84 (0.76, 0.92)** **	
F test *P* value (*df*)	.97 (1)	.3 (1)	**.02 (1)**	**<.001 (1)**	**.02 (3)**

Bold text indicates a statistically significant difference with a *p*‐value less than .05.

^a^Survey weighted models include ethnicity, all socioeconomic variables, sex, country, urban/rural, and number of permanent and primary teeth.

^b^Three indicators (presence of plaque or calculus in more than 1 sextant, gingivitis) were combined to produce an indicator of poor periodontal health.

*P < .05; **P < .01; ***P< .001.

At age 5 and 8, there was strong evidence of ethnic and SEP differences in tooth decay (Table 2). The predicted rate of decayed teeth for White British/Irish children aged 5 was 1.54 (95%CI 1.30‐1.77). In contrast, the predicted rate for Indian and Pakistani children was about 2‐2.5 times higher. The predicted rate of decay was lowest among Black African children at age 5. Furthermore, Black African children tended to have lower levels of decay at all ages. In contrast to the pattern of ethnic differences in decay at age 5 and 8, there was very weak evidence of ethnic differences in decay among children aged 12 and 15.

Turning to socioeconomic differences, poorer family SEP (free school meal eligibility) and greater area deprivation (IMD rank) were associated with higher rates of decay among children age 5, and 12, while at age 15 none of the SEP measures appeared to influence decay.

In terms of ethnic differences in filled teeth for children at age 5 (Table 3), other White children had higher predicted rates of filled teeth compared to the White British/Irish children. However, ethnic minority children at age 15 did not have significantly higher rates of filled teeth compared to White British/Irish children. Indeed, 15‐year‐old Bangladeshi children had significantly lower rates of filled teeth compared to White British/Irish children. There was little evidence for socioeconomic differences in filled teeth except for children aged 15 living in deprived areas.

Poor periodontal health was associated with ethnicity among children aged 5 and 12 (Table 4), with Bangladeshi children having the highest predicted probabilities of poor periodontal health. Bangladeshi children aged 5 also had the highest predicted probabilities of gingivitis (Table S7) and plaque (Table S8). In contrast, at age 15, there were no significant ethnic differences in poor periodontal health, gingivitis and plaque. There was no evidence of socioeconomic differences in periodontal health, gingivitis and plaque, except at ages 12 and 15, when adolescents from deprived areas (higher IMD rank) had higher probabilities of poor oral health on all these 3 measures.

In Tables 2, 3, 4 and S6‐S8, we examined whether the differences in our selected oral health outcomes by SEP/ethnicity across children's age were statistically significant. The interaction term between ethnicity and age was significant for tooth decay (Table 2), filled teeth (Table 3) and plaque (Table S8), suggesting that the risk of poor oral health for ethnic minority children significantly reduces from age 5 to age 15. Figure S1 shows the predicted rate of tooth decay among children of White, other White, Indian and Pakistani ethnicity. The ethnic differences that are clearly shown among children aged 5 are no longer apparent among children aged 15.

The interaction term between free school meal and age was also statistically significant for tooth decay (Table 2), suggesting that the higher levels of tooth decay among free school meal eligible children aged 5 had reduced considerably among children aged 15. No significant interactions between IMD rank and age were found, with the exception of poor periodontal health (Table 4). This suggests that the association between deprivation and poor oral health remains the same across different children's ages, and in the case of poor periodontal health, this association was stronger among older children.

We calculated the relative index of inequality (RII) based on the IMD rank for children living in each country for the 5 measures of oral health (Table S9). The RII for gingivitis, plaque and poor periodontal health was largest for 15‐year‐old children living in England, Wales and Northern Ireland compared to younger children from the same country. In contrast, the RII for tooth decay and filled teeth tended to be smaller among children aged 15 from each country compared to 5‐year‐old children from the same country. There were no significant interactions between the RII and country, with the exception of 12‐year‐old children. Children living in England had higher relative inequalities in tooth decay and poor periodontal health than children living in Wales and Northern Ireland.

We also examined whether the association between ethnicity and each of the oral health measures reduced in the regression models after controlling for the SEP measures. While the pattern of the association was similar in the models with and without controlling for SEP, there was a decrease in the size of the coefficients for Pakistani, Bangladeshi after controlling for SEP, reflecting the disadvantaged socioeconomic circumstances of the 5‐year‐old children from those ethnic groups.

## 4 DISCUSSION

We found strong evidence of smaller ethnic differences in dental decay among British adolescents aged 15 compared to children aged 5, and this pattern was repeated for all the oral health measures. Moreover, the socioeconomic gap (using free school meal eligibility) in dental decay among children was significantly smaller among children aged 15 compared to 5‐year‐old children. However, the association between higher levels of residential deprivation and higher levels of dental decay remained similar across all the age groups. Furthermore, the association between residential deprivation and poor periodontal health was stronger among 15‐year‐old children compared to 5‐year‐old children.

The equalization hypothesis suggests that socioeconomic inequalities in health reduce as children age.^3^ Our study on children and adolescent oral health showed a complex picture, which does not fully support this hypothesis. There was some evidence of equalization in terms of ethnic and family‐based SEP differences, particularly in terms of dental decay, but differences by residential deprivation remained throughout childhood and adolescence for dental decay, and increased during adolescence for filled teeth and poor periodontal health.

Although the hypothesis of socioeconomic equalization in oral health during adolescence has not been explicitly examined in previous studies, there is some cross‐sectional evidence of smaller ethnic differences in oral health among Danish adolescents^28^ and smaller socioeconomic differences among US^18^ and French^29^ adolescents, compared to younger children. Moreover, using longitudinal data from New Zealand, Lewsey and Thomson^19^ showed that the substantial SEP differences, which existed at age 5 (in the primary dentition), had reduced somewhat by age 18 and had widened again by age 26.

Late childhood and early adolescence is an important stage of the lifecourse, partly because it represents the transition from a more circumscribed, family centred environment, to a broader environment more open to influences of peers and nonfamily members.^30^ The school environment could contribute to the social equalization of the health of adolescents but creates at the same time new disparities, with probably long‐lasting consequences.^4^ In our study, residential deprivation predicted poorer oral health among adolescents, whereas among younger children, it was family‐based SEP that predicted tooth decay. Children who live in more deprived areas are closer to fast food outlets than children in less deprived areas.^31^ Area characteristics of the food environment have been shown to be associated with weight‐status of children living in England. Compared to 4‐5 year‐old children, the association between children's weight and area characteristics was greater for 10‐11 year‐old children who have more independence in their purchasing decision than younger children.^32^ Moreover, as adolescents may purchase food and drinks in and around their schools, the school food environment may also be important for oral health‐related eating behaviours such as soft drink and snack consumption.

Oral health behaviours change during adolescence. Very young children's tooth brushing behaviours are often supervised by their parents,^33^ whereas adolescents brush their teeth with varying degrees of skill or commitment. Moreover, peer group and mass media become more relevant during adolescence.^34^, ^35^ The availability and consumption of sugary food and drinks is greater in adolescence.^34^ Alongside these changes in oral health behaviours during adolescence, there are changes in the ethnic and socioeconomic differences in these behaviours. White British adolescents have higher levels of risky health behaviours compared to ethnic minority adolescents.^36^ The socioeconomic gap in oral health promoting behaviours such as toothbrushing increases among adolescents aged 15 compared to those aged 12. Moreover, the socioeconomic gap in drinking water actually reverses as children grow older. Drinking water is more common among disadvantaged adolescents aged 12, whereas the advantaged adolescents aged 15 are more likely to drink water.^37^

In the UK, marked socioeconomic and ethnic inequalities exist for the use of dental services.^38^ All the major ethnic minority and disadvantaged socioeconomic groups are less likely to visit the dentist, and more likely to visit due to problems with their teeth.^25^ In our study, we found that adjusting for multiple measures of socioeconomic disadvantage did not explain the poorer oral health among ethnic minority children aged 5 and 8. Cultural beliefs play a role in dental care seeking behaviour. Due to the transient nature of the primary teeth, some caregivers of young children feel their care are not as important as permanent teeth and some cultural and ethnic groups may not have a strong preventive oral health orientation.^39^, ^40^

The type and variety of foods consumed in the early years influence longer‐term eating behaviours.^41^ Although White mothers report that they introduce solid foods earlier than mothers of Asian origin,^42^ they were more likely to consider the sugar content of their child's food and to avoid teeth‐damaging foods, whereas Pakistani mothers have been found to be more likely to give sweetened drinks and foods at an early age.^42^

The main limitation of this study is that the data came from a cross‐sectional survey. We were not able to examine how socioeconomic and ethnic inequalities in oral health changed as the children grew into adolescents. Hence, any differences in oral health between adolescents and children may not be related to the lifecourse and ageing, but may reflect cohort differences. Due to small numbers of cases from certain ethnic groups, we had to drop these groups from the main analyses. We acknowledge the risks that more complex empirical patterns might be overlooked. This was explored further in the sensitivity analyses (Table S10). Another limitation refers to the conceptual and methodological adequacy of the SEP measures. Free school meal eligibility is a binary measure and does not capture the dimensional nature of socioeconomic disadvantage. Social class or parents' educational level may be better at reflecting other dimension of socioeconomic disadvantage but they were not assessed in the survey.

This study found some evidence of smaller ethnic and family socioeconomic differences in oral health among British adolescents compared to younger children. However, contrary to the equalization in adolescence hypothesis, substantial differences in oral health by residential deprivation remain among adolescent children. This study underlines the importance of using multiple measures of socioeconomic disadvantage when analysing oral health inequalities. Community levels of deprivation may be particularly important for the health of adolescents.
