# Supplementary material for: Socioeconomic and ethnic inequalities in oral health among children and adolescents living in England, Wales and Northern Ireland
Source: Community Dent Oral Epidemiol. 2018 Jun 10;46(5):426–34. doi: 10.1111/cdoe.12390 (PMC6849874; doi:10.1111/cdoe.12390)
Supplement: Supplementary file 1 [file CDOE-46-426-s001.docx]

**Supporting Information**

**Content:**

Methods. Additional Description of the Methodology

Descriptive statistics Appendix Tables S1 to S4

Sensitivity Analyses

References

**List of Appendix Tables and Figure**

**Table S1.** Mean (sd) number of teeth and oral health characteristics (%) by ethnicity and socioeconomic position: children aged 5, N=2,217 (CDHS 2013)

**Table S2.** Mean (sd) number of teeth and oral health characteristics (%) by ethnicity and socioeconomic position: children aged 8, N=2,083 (CDHS 2013)

**Table S3.** Mean (sd) number of teeth and oral health characteristics (%) by ethnicity and socioeconomic position: children aged 12, N=2,183 (CDHS 2013)

**Table S4.** Mean (sd) number of teeth and oral health characteristics (%) by ethnicity and socioeconomic position: children aged 15, N=2,058 (CDHS 2013)

**Table S5.** Weighted percentages of children with clinical decay (including visual and cavitated enamel) criteria 2013; and obvious decay (visual and cavitated dentine) criteria 2003 (CDHS 2013)

**Table S6.** Predicted Rates (PRs) and 95% Confidence Intervals (CIs) from negative binomial regression models^a,b^: obvious tooth decay (2003 criteria) in children regressed on ethnicity/SEP in the four age-cohort samples (CDHS 2013)

**Table S7.** Predicted Probabilities (PPs) and 95% Confidence Intervals (CIs) from probit regression models^a^: gingivitis in children regressed on ethnicity/SEP in the four age-cohort samples (CDHS 2013)

**Table S8.** Predicted Probabilities (PPs) and 95% Confidence Intervals (CIs) from probit regression models^a^: plaque in children regressed on ethnicity/SEP in the four age-cohort samples (CDHS 2013)

**Table S9.** Relative Index of Inequality (RII, from ranked Index of Multiple Deprivation) for tooth decay, filled teeth, plaque, gingivitis and poor periodontal health, by country, with p-values for the interaction term between RII and country

**Table S10.** Odds ratios (ORs) and 95% Confidence Intervals (CIs) of decay in primary teeth of children aged 5 and permanent teeth of children aged 15, by ethnicity and socioeconomic position (CDHS 2013): Multilevel model of teeth (level 1) clustered within children (level 2)

**Figure S1.** Predicted Rates and 95% Confidence Intervals of tooth decay by White British/Irish, Other White, Indian and Pakistani ethnic groups: children aged 5 and 15 (from Table 2)

**Methods.** Additional Description of the Methodology

In this supplement, further descriptions of the variables related to oral health, socioeconomic position, ethnicity and covariates are detailed. We also describe additional sensitivity analyses to test the robustness of the associations reported in the main analyses.

**Oral Health**

Thresholds for detecting tooth decay: The data on tooth decay used in the study reflect the context of changes in diagnostic criteria. Since 1973, the diagnosis thresholds used for caries detection has evolved from a cavitated lesion into dentine to include a more complete assessment allowing the detection of caries at an earlier visual stage. In 2003, in addition of cavitated lesions into dentine, the caries detection incorporated visual lesion into dentine, termed obvious decay. It has been the traditional measure used in dental epidemiology surveys seeking to establish the number of “cavities” to be “filled”. In 2013 enamel caries both at a visual and cavitated stage was also recorded, termed clinical decay. Enamel decay does not usually require a filling but may indicate the need for interventions to prevent decay progressing into dentine. A more detail illustration of the terminology and criteria for dental decay can be found here: <http://content.digital.nhs.uk/catalogue/PUB17137/CDHS2013-Report2-Dental-Disease.pdf>

The International Caries Detection Assessment System has been recognized as a valuable method for detecting the enamel caries lesions for planning the individual remineralization therapy by fluorides or for monitoring the caries pattern at the population level.

For each age-cohort, teeth were examined for dental caries and restorations, with 4 surfaces (buccal, lingual, distal, and mesial) being considered for canines and incisors, and a fifth surface (the occlusal) included for premolars and molars. In addition to the tooth dental examination data, we also used tooth surface dental examination data. Only findings from tooth dental examination data are shown.

**Periodontal disease**

The measures that were used for visual assessment of gum condition were the presence or absence of risk factors for gum disease - plaque and calculus - and whether the gingivae, the soft gum tissue, appeared healthy or not. The assessment was made for each sextant in the child’s mouth (both the upper arch and lower arch of the mouth can be split into three sextants – so in the case of the upper arch of the mouth, this would be the upper right, upper central and upper left sextants).

**Demographic variables**

Ethnicity: In the UK the concepts of race and ethnicity are intertwined. Ethnicity refers to a sense of belonging to a group of people who share characteristics such as cultural values, language, religion, history, and skin color. Further, the UK census questions use the term ‘ethnic group’ rather than ‘race’.^1^ Thus throughout the manuscript we use the term ‘ethnicity’ rather than ‘race/ethnicity’. Ethnicity of the children was collected from school records, which used parents’ reporting of family ethnic group when their child started at school. The 2011 census ethnic categories were used^2^. The 8 ethnic groups used for analysis were: White British/Irish; other White background; White & Black Caribbean / African; White & Asian; other Mixed background; Indian; Pakistani; Bangladeshi; Black African; Black Caribbean / other Black-African-Caribbean. For the main analyses, due to small numbers of cases and heterogeneous groups, we excluded other ethnic groups, including other Asian; Chinese; Arab; Gypsy and Irish travellers; and other unspecified ethnic group. The other White children in the CDHS largely represent children of Eastern European origin,^2^ as the survey over sampled schools in deprived areas where Eastern European origin families are more likely to live. There was no other information on other aspects of ethnicity such as country of birth, language, nationality, or religion. The use of multiple measures, which examine different aspects of the concept of ethnicity would have offer a more effective approach to the measurement of ethnicity in more detail,

Rural and urban areas were classified by the Office for National Statistics Output Area Classification (OAC). This classification groups small areas based on similarities across a variety of 2011 census indicators covering demographic composition, household composition, housing, socio-economic status and employment.^4^

The Index of Multiple Deprivation (IMD) is a measure of relative deprivation for small areas (or Lower Super Output Areas, which are fixed statistical geographies of about 1,500 people). It is a combined measure of deprivation based on separate indicators that have been grouped into domains (income, employment, health, education, barriers to housing and services, community safety, and living environment), each of which reflects a different aspect of deprivation experienced by individuals living in an area. The latest index for each country was used at the time of linking, which was the 2010 Index for England;^6^ the 2011 index for Wales;^7^ and the 2010 Northern Ireland Multiple Deprivation Measure.^8^ However, due to the fact that the constituent nations of the UK have constructed somewhat different indices, direct comparisons between the countries on the basis of these indices is not possible. Using country-specific IMD for between-country comparisons may give misleading results with respect to health outcomes.

**Descriptive statistics Appendix Tables S1 to S4**

**Table S1.** Survey weighted mean (sd) number of teeth and oral health characteristics (%) by ethnicity and socioeconomic position: children aged 5, N=2,217 (CDHS 2013)

| **Variables** | **mean (sd): teeth with decay** | **mean (sd): teeth with fillings** | **mean (sd): primary teeth** | **mean (sd): permanent teeth** | **Gingivitis %** | **Plaque %** | **Poor periodontal health %** |
| --- | --- | --- | --- | --- | --- | --- | --- |
| **Ethnicity** |  |  |  |  |  |  |  |
| White British & Irish | 1.48 (2.46) | 0.09 (0.45) | 18.98 (1.80) | 1.58 (2.39) | 23.3% | 32.0% | 43.9% |
| Other White | 2.11 (2.19) | 0.30 (0.71) | 17.85 (2.71) | 2.31 (2.24) | 24.8% | 25.8% | 41.3% |
| Mixed White | 1.93 (2.44) | 0.08 (0.28) | 18.45 (1.86) | 1.87 (2.36) | 27.2% | 47.0% | 60.1% |
| Indian | 2.83 (2.52) | 0.17 (0.39) | 19.13 (1.33) | 1.71 (1.92) | 26.3% | 31.8% | 49.9% |
| Pakistani | 3.04 (3.51) | 0.18 (0.55) | 18.47 (2.61) | 1.91 (2.48) | 25.1% | 50.8% | 58.1% |
| Bangladeshi | 2.52 (2.77) | 0.20 (0.79) | 19.15 (2.04) | 1.13 (1.82) | 42.2% | 56.8% | 69.6% |
| Black African | 0.81 (1.20) | 0.31 (0.96) | 17.46 (1.93) | 3.12 (2.77) | 11.9% | 25.4% | 29.4% |
| Black Caribbean | 1.65 (1.52) | 0.04 (0.21) | 17.82 (1.35) | 3.09 (2.03) | 15.4% | 27.0% | 28.7% |
| **Free school meal** |  |  |  |  |  |  |  |
| not eligible | 1.44 (2.19) | 0.12 (0.49) | 18.83 (1.91) | 1.73 (2.32) | 22.1% | 31.8% | 43.8% |
| eligible | 2.39 (3.50) | 0.11 (0.58) | 18.65 (2.34) | 1.75 (2.86) | 29.4% | 37.3% | 47.7% |
| **Deprived school** |  |  |  |  |  |  |  |
| no | 1.44 (2.07) | 0.11 (0.44) | 18.86 (1.77) | 1.66 (2.17) | 22.2% | 31.4% | 43.6% |
| yes | 2.27 (3.86) | 0.15 (0.75) | 18.52 (2.79) | 2.03 (3.33) | 28.1% | 38.2% | 47.6% |
| **Index of Multiple Deprivation quintiles (home postcode)** | | | | |  |  |  |
| **England n=1,279** |  |  |  |  |  |  |  |
| least deprived | 1.22 (1.62) | 0.08 (0.30) | 18.98 (1.42) | 1.77 (1.96) | 19.6% | 35.6% | 47.5% |
| Quintile 2 | 0.88 (1.47) | 0.04 (0.25) | 18.91 (1.48) | 1.67 (1.98) | 16.8% | 27.1% | 46.6% |
| Quintile 3 | 1.34 (2.01) | 0.11 (0.40) | 19.00 (1.53) | 1.48 (2.01) | 19.8% | 27.0% | 40.5% |
| Quintile 4 | 1.83 (2.65) | 0.11 (0.56) | 18.62 (2.39) | 1.86 (2.70) | 25.2% | 34.7% | 39.9% |
| most deprived | 2.27 (3.48) | 0.15 (0.70) | 18.59 (2.64) | 1.93 (3.06) | 31.1% | 35.7% | 47.5% |
| **Wales n=425** |  |  |  |  |  |  |  |
| least deprived | 1.07 (1.07) | 0.05 (0.21) | 18.81 (1.98) | 1.23 (1.86) | 42.6% | 59.4% | 46.1% |
| Quintile 2 | 1.69 (2.20) | 0.29 (0.94) | 18.83 (1.55) | 1.71 (1.91) | 24.9% | 26.5% | 39.3% |
| Quintile 3 | 2.01 (2.74) | 0.09 (0.35) | 18.84 (1.70) | 1.17 (1.50) | 28.9% | 61.1% | 66.3% |
| Quintile 4 | 2.50 (3.04) | 0.31 (0.91) | 18.56 (2.25) | 1.5 (2.55) | 20.8% | 36.6% | 32.1% |
| most deprived | 2.43 (3.60) | 0.26 (0.88) | 18.65 (2.80) | 1.51 (3.23) | 28.9% | 40.3% | 60.7% |
| **Northern Ireland n=513** | |  |  |  |  |  |  |
| least deprived | 0.94 (1.63) | 0.12 (0.57) | 19.12 (1.51) | 1.56 (2.37) | 17.2% | 28.6% | 47.1% |
| Quintile 2 | 1.35 (2.24) | 0.15 (0.54) | 18.59 (2.16) | 1.74 (2.68) | 26.6% | 37.4% | 34.5% |
| Quintile 3 | 1.47 (2.43) | 0.17 (0.74) | 18.37 (2.64) | 1.87 (2.54) | 9.9% | 24.0% | 27.4% |
| Quintile 4 | 2.27 (3.28) | 0.25 (1.03) | 18.68 (2.22) | 1.81 (2.79) | 19.0% | 33.7% | 44.4% |
| most deprived | 3.49 (2.72) | 0.43 (0.77) | 18.77 (1.78) | 1.91 (2.39) | 17.7% | 45.5% | 33.9% |

**Table S2.** Survey weighted mean (sd) number of teeth and oral health characteristics (%) by ethnicity and socioeconomic position: children aged 8, N=2,083 (CDHS 2013)

| **Variables** | **mean (sd): teeth with decay** | **mean (sd): teeth with fillings** | **mean (sd): primary teeth** | **mean (sd): permanent teeth** | **Gingivitis %** | **Plaque %** | **Poor periodontal health %** |
| --- | --- | --- | --- | --- | --- | --- | --- |
| **Ethnicity** |  |  |  |  |  |  |  |
| White British & Irish | 2.17 (2.74) | 0.37 (0.97) | 11.29 (2.47) | 11.93 (2.19) | 50.0% | 56.0% | 71.4% |
| Other White | 3.39 (2.68) | 0.47 (0.70) | 10.66 (1.99) | 12.43 (1.76) | 53.5% | 63.7% | 69.3% |
| Mixed White | 2.77 (2.68) | 0.37 (0.74) | 11.30 (2.19) | 12.14 (2.00) | 42.8% | 69.3% | 83.1% |
| Indian | 3.25 (3.34) | 0.58 (1.14) | 11.09 (2.21) | 12.28 (1.12) | 62.7% | 57.9% | 75.3% |
| Pakistani | 3.89 (3.56) | 0.64 (1.26) | 9.67 (3.31) | 12.29 (2.24) | 49.7% | 63.8% | 78.0% |
| Bangladeshi | 2.79 (2.39) | 0.35 (0.84) | 11.01 (2.84) | 11.47 (2.46) | 41.6% | 61.6% | 75.8% |
| Black African | 1.40 (1.46) | 0.79 (1.14) | 9.87 (2.83) | 13.71 (2.97) | 10.5% | 49.4% | 71.2% |
| Black Caribbean | 1.96 (1.71) | 0.14 (0.45) | 10.84 (2.42) | 12.68 (2.04) | 24.2% | 49.9% | 60.7% |
| **Free school meal** |  |  |  |  |  |  |  |
| not eligible | 2.21 (2.62) | 0.39 (0.93) | 11.25 (2.38) | 12.05 (2.19) | 48.1% | 56.9% | 72.0% |
| eligible | 2.81 (3.41) | 0.46 (1.09) | 10.60 (3.29) | 12.05 (2.57) | 46.9% | 57.7% | 70.5% |
| **Deprived school** |  |  |  |  |  |  |  |
| no | 2.20 (2.50) | 0.37 (0.85) | 11.24 (2.24) | 12.04 (2.05) | 47.6% | 56.4% | 71.0% |
| yes | 2.68 (3.59) | 0.50 (1.35) | 10.79 (3.57) | 12.08 (2.95) | 49.2% | 58.5% | 74.9% |
| **Index of Multiple Deprivation quintiles (home postcode)** | | | | |  |  |  |
| **England n=1,163** |  |  |  |  |  |  |  |
| least deprived | 1.70 (2.03) | 0.23 (0.55) | 11.45 (1.79) | 11.97 (1.72) | 54.9% | 60.4% | 78.5% |
| Quintile 2 | 1.82 (0.38) | 0.39 (0.70) | 11.49 (2.28) | 11.92 (2.21) | 43.8% | 60.8% | 71.9% |
| Quintile 3 | 1.96 (2.07) | 0.30 (0.69) | 11.48 (1.78) | 11.93 (1.46) | 44.0% | 48.5% | 64.8% |
| Quintile 4 | 2.44 (2.75) | 0.57 (1.33) | 11.28 (2.62) | 11.78 (2.32) | 44.2% | 55.6% | 68.2% |
| most deprived | 2.93 (3.49) | 0.41 (1.23) | 10.55 (3.44) | 12.38 (3.09) | 51.3% | 57.4% | 74.9% |
| **Wales n=429** |  |  |  |  |  |  |  |
| least deprived | 3.34 (2.49) | 0.32 (0.41) | 11.56 (0.96) | 12.07 (0.96) | 75.6% | 71.6% | 79.6% |
| Quintile 2 | 2.61 (2.93) | 0.48 (1.15) | 11.08 (2.29) | 12.36 (2.16) | 39.9% | 64.0% | 72.4% |
| Quintile 3 | 3.66 (4.27) | 0.41 (1.03) | 11.37 (1.68) | 12.04 (1.34) | 49.1% | 75.8% | 89.1% |
| Quintile 4 | 4.05 (3.92) | 0.30 (0.96) | 10.75 (3.10) | 12.14 (2.52) | 51.5% | 68.6% | 78.6% |
| most deprived | 2.69 (3.96) | 0.54 (1.06) | 10.75 (3.25) | 12.02 (2.13) | 57.2% | 65.9% | 77.3% |
| **Northern Ireland n=491** | |  |  |  |  |  |  |
| least deprived | 2.06 (2.03) | 0.51 (0.80) | 10.58 (2.53) | 12.50 (2.34) | 40.2% | 63.5% | 71.9% |
| Quintile 2 | 2.14 (2.82) | 0.75 (1.32) | 11.03 (2.23) | 12.14 (2.05) | 32.1% | 64.1% | 70.6% |
| Quintile 3 | 2.51 (3.05) | 0.60 (1.12) | 10.30 (2.88) | 12.14 (1.99) | 35.8% | 43.5% | 54.9% |
| Quintile 4 | 1.59 (2.31) | 0.58 (1.16) | 10.10 (3.10) | 12.55 (2.44) | 37.0% | 39.7% | 47.4% |
| most deprived | 2.85 (2.00) | 0.67 (0.99) | 9.72 (2.95) | 12.91 (2.35) | 53.6% | 74.2% | 77.7% |

**Table S3.** Survey weighted mean (sd) number of teeth and oral health characteristics (%) by ethnicity and socioeconomic position: children aged 12, N=2,183 (CDHS 2013)

| **Variables** | **mean (sd): teeth with decay** | **mean (sd): teeth with fillings** | **mean (sd): primary teeth** | **mean (sd): permanent teeth** | **Gingivitis %** | **Plaque %** | **Poor periodontal health %** |
| --- | --- | --- | --- | --- | --- | --- | --- |
| **Ethnicity** |  |  |  |  |  |  |  |
| White British & Irish | 1.74 (2.75) | 0.40 (0.88) | 1.06 (2.26) | 25.35 (3.74) | 61.3% | 52.8% | 73.1% |
| Other White | 2.21 (2.43) | 0.51 (0.85) | 0.59 (1.44) | 25.94 (2.48) | 76.2% | 61.8% | 77.0% |
| Mixed White | 1.23 (1.79) | 0.44 (0.78) | 0.85 (1.92) | 25.6 (3.33) | 63.3% | 55.4% | 76.1% |
| Indian | 1.20 (2.63) | 0.26 (0.49) | 0.81 (1.67) | 25.89 (2.90) | 56.3% | 43.8% | 75.3% |
| Pakistani | 1.83 (2.46) | 0.61 (1.41) | 0.76 (1.70) | 24.83 (4.24) | 66.0% | 46.3% | 79.8% |
| Bangladeshi | 1.23 (2.18) | 0.22 (0.60) | 0.33 (1.42) | 26.56 (2.67) | 58.9% | 69.6% | 93.5% |
| Black African | 0.76 (1.29) | 0.25 (0.60) | 0.39 (1.18) | 26.10 (2.23) | 47.8% | 43.7% | 72.1% |
| Black Caribbean | 1.48 (1.94) | 0.32 (0.68) | 0.16 (0.36) | 26.61 (1.68) | 66.4% | 71.3% | 82.5% |
| **Free school meal** |  |  |  |  |  |  |  |
| not eligible | 1.48 (2.36) | 0.38 (0.83) | 0.95 (2.03) | 25.52 (3.42) | 60.9% | 52.1% | 74.1% |
| eligible | 2.39 (3.48) | 0.47 (1.06) | 0.97 (2.40) | 25.27 (4.15) | 63.9% | 58.7% | 76.2% |
| **Deprived school** |  |  |  |  |  |  |  |
| no | 1.72 (2.42) | 0.41 (0.80) | 1.10 (2.00) | 25.42 (3.23) | 60.7% | 52.0% | 72.0% |
| yes | 1.54 (3.03) | 0.35 (1.06) | 0.45 (1.94) | 25.63 (4.48) | 64.5% | 58.7% | 83.1% |
| **Index of Multiple Deprivation quintiles (home postcode)** | | | | |  |  |  |
| **England n=1,220** |  |  |  |  |  |  |  |
| least deprived | 1.03 (1.61) | 0.27 (0.63) | 0.95 (2.12) | 25.54 (3.20) | 57.5% | 48.3% | 70.7% |
| Quintile 2 | 1.22 (2.03) | 0.40 (0.85) | 1.22 (2.04) | 25.18 (3.35) | 56.8% | 46.5% | 67.6% |
| Quintile 3 | 1.75 (2.64) | 0.35 (0.70) | 1.10 (2.22) | 25.64 (3.46) | 52.8% | 55.1% | 63.1% |
| Quintile 4 | 1.31 (1.75) | 0.40 (0.83) | 1.13 (2.22) | 24.95 (3.95) | 66.9% | 59.2% | 80.9% |
| most deprived | 2.24 (3.59) | 0.37 (0.91) | 0.75 (1.97) | 25.73 (3.68) | 69.5% | 55.9% | 81.8% |
| **Wales n=539** |  |  |  |  |  |  |  |
| least deprived | 1.48 (1.45) | 0.48 (0.73) | 1.38 (2.21) | 24.52 (3.37) | 69.6% | 48.1% | 84.1% |
| Quintile 2 | 2.01 (2.30) | 0.64 (0.98) | 0.38 (0.90) | 26.75 (1.60) | 42.4% | 52.1% | 66.5% |
| Quintile 3 | 2.04 (2.38) | 0.49 (1.03) | 0.66 (1.71) | 25.91 (2.79) | 57.3% | 47.0% | 64.6% |
| Quintile 4 | 2.70 (2.77) | 0.48 (0.86) | 0.88 (1.88) | 25.17 (3.18) | 42.4% | 64.4% | 74.8% |
| most deprived | 3.02 (3.5) | 0.72 (1.31) | 0.56 (1.94) | 26.27 (3.21) | 54.4% | 60.7% | 73.6% |
| **Northern Ireland n=424** | |  |  |  |  |  |  |
| least deprived | 2.87 (1.98) | 0.65 (1.00) | 1.17 (1.59) | 24.81 (2.46) | 37.6% | 64.1% | 81.0% |
| Quintile 2 | 1.91 (2.75) | 0.98 (1.45) | 0.70 (1.42) | 25.93 (2.66) | 51.1% | 51.9% | 65.7% |
| Quintile 3 | 0.81 (1.47) | 0.69 (1.23) | 0.72 (1.86) | 25.67 (3.41) | 45.3% | 44.6% | 55.7% |
| Quintile 4 | 1.83 (2.61) | 1.19 (1.51) | 0.43 (1.31) | 25.61 (3.40) | 45.1% | 43.7% | 56.0% |
| most deprived | 2.00 (3.38) | 1.37 (2.01) | 0.32 (1.48) | 26.14 (2.68) | 48.8% | 46.3% | 66.6% |

**Table S4.** Survey weighted mean (sd) number of teeth and oral health characteristics (%) by ethnicity and socioeconomic position: children aged 15, N=2,058 (CDHS 2013)

| **Variables** | **mean (sd): teeth with decay** | **mean (sd): teeth with fillings** | **mean (sd): primary teeth** | **mean (sd): permanent teeth** | **Gingivitis %** | **Plaque %** | **Poor periodontal health %** |
| --- | --- | --- | --- | --- | --- | --- | --- |
| **Ethnicity** |  |  |  |  |  |  |  |
| White British & Irish | 1.83 (2.92) | 0.83 (1.55) | 0.09 (0.50) | 27.20 (1.59) | 54.0% | 39.3% | 67.0% |
| Other White | 1.47 (2.67) | 0.99 (1.34) | 0.04 (0.17) | 27.48 (1.03) | 42.5% | 28.8% | 68.5% |
| Mixed White | 2.18 (2.15) | 1.23 (1.34) | 0.09 (0.27) | 26.94 (1.08) | 45.1% | 35.4% | 70.6% |
| Indian | 1.18 (1.53) | 0.58 (1.24) | 0.01 (0.10) | 27.52 (1.08) | 58.5% | 33.9% | 60.4% |
| Pakistani | 1.99 (3.18) | 0.62 (1.36) | 0.01 (0.09) | 27.14 (1.66) | 74.2% | 40.4% | 81.7% |
| Bangladeshi | 1.73 (2.27) | 0.37 (0.71) | 0.04 (0.23) | 27.49 (1.20) | 56.3% | 46.0% | 80.4% |
| Black African | 1.95 (2.47) | 0.39 (0.83) | 0.00 (0.00) | 27.56 (1.02) | 78.8% | 46.5% | 86.7% |
| Black Caribbean | 1.82 (2.44) | 0.57 (0.94) | 0.09 (0.32) | 27.57 (1.07) | 58.4% | 28.3% | 73.5% |
| **Free school meal** |  |  |  |  |  |  |  |
| not eligible | 1.74 (2.68) | 0.77 (1.39) | 0.07 (0.39) | 27.26 (1.38) | 53.0% | 36.8% | 66.5% |
| eligible | 2.21 (3.47) | 1.01 (1.93) | 0.13 (0.75) | 27.10 (2.11) | 63.0% | 48.6% | 77.6% |
| **Deprived school** |  |  |  |  |  |  |  |
| no | 1.75 (2.39) | 0.79 (1.29) | 0.09 (0.43) | 27.21 (1.36) | 52.3% | 37.0% | 65.8% |
| yes | 2.05 (4.19) | 0.86 (2.02) | 0.04 (0.34) | 27.31 (1.76) | 63.0% | 45.2% | 77.7% |
| **Index of Multiple Deprivation quintiles (home postcode)** | | | | |  |  |  |
| **England n=1,123** |  |  |  |  |  |  |  |
| least deprived | 1.50 (2.78) | 0.43 (1.12) | 0.09 (0.48) | 27.44 (1.09) | 43.7% | 27.1% | 61.3% |
| Quintile 2 | 1.66 (2.41) | 0.62 (1.28) | 0.07 (0.28) | 27.30 (1.15) | 49.7% | 30.9% | 57.7% |
| Quintile 3 | 1.42 (2.25) | 0.66 (1.04) | 0.14 (0.73) | 27.17 (1.76) | 43.7% | 31.4% | 59.6% |
| Quintile 4 | 1.58 (2.54) | 0.99 (1.41) | 0.07 (0.28) | 27.09 (1.45) | 55.9% | 45.3% | 73.5% |
| most deprived | 2.23 (3.24) | 0.81 (1.53) | 0.06 (0.35) | 27.28 (1.47) | 70.1% | 47.0% | 80.7% |
| **Wales n=464** |  |  |  |  |  |  |  |
| least deprived | 1.94 (2.37) | 1.12 (2.22) | 0.00 (0.00) | 27.35 (1.17) | 37.6% | 28.5% | 59.3% |
| Quintile 2 | 2.06 (2.42) | 1.01 (1.63) | 0.02 (0.11) | 26.99 (1.31) | 39.2% | 46.8% | 66.5% |
| Quintile 3 | 1.92 (2.73) | 1.48 (2.04) | 0.04 (0.27) | 26.27 (4.53) | 51.4% | 38.9% | 63.2% |
| Quintile 4 | 2.95 (2.74) | 0.98 (1.25) | 0.10 (0.43) | 26.90 (1.50) | 36.9% | 52.8% | 68.2% |
| most deprived | 2.39 (3.30) | 1.45 (2.23) | 0.03 (0.23) | 27.24 (1.66) | 56.8% | 54.9% | 79.1% |
| **Northern Ireland n=471** | |  |  |  |  |  |  |
| least deprived | 2.44 (2.63) | 1.83 (3.58) | 0.03 (0.16) | 27.29 (1.15) | 43.2% | 47.5% | 62.4% |
| Quintile 2 | 1.80 (3.07) | 1.28 (1.66) | 0.06 (0.44) | 27.33 (1.34) | 48.3% | 42.3% | 52.4% |
| Quintile 3 | 1.58 (3.98) | 2.57 (2.78) | 0.04 (0.26) | 27.10 (1.35) | 36.4% | 31.1% | 47.5% |
| Quintile 4 | 1.74 (2.81) | 2.43 (2.41) | 0.19 (0.79) | 27.05 (1.56) | 38.9% | 34.4% | 45.7% |
| most deprived | 2.75 (4.93) | 2.57 (3.06) | 0.01 (0.11) | 26.74 (1.72) | 53.2% | 45.4% | 70.2% |

**Sensitivity Analyses** The following sensitivity analyses were carried out.

*1. Sex differences:* There was little evidence of sex differences in the oral health outcome measures, with the exception of poor periodontal health at ages 12 and 15. Girls were less likely to have poor periodontal health compared to boys at those ages. We also examined if there were any significant sex differences in the associations between ethnicity, SEP and the oral health measures but did not find strong evidence for such sex interactions with ethnicity and SEP.

*2. Tooth-surface dental examination data:* In the negative binomial regression models predicting tooth decay and filled teeth, we created additional dependent variables that were the number of surfaces with decay and the number of surfaces with fillings. We compared the coefficients of ethnicity and SEP from these models to the models where the dependent variables were the number of teeth with decay and fillings (Tables 2 and 3 in the main manuscript). The coefficients hardly changed, indicating that examining decay or fillings using the tooth surface data (rather than the tooth data) did not change the reported associations between ethnicity/SEP and decayed/filled teeth.

*3. 2003 criteria:* In addition to the 2013 criteria for recording dental decay, we used the 2003 criteria, which does not include visual and cavitated decay into enamel. Table S5 below, we show how the prevalence of children with signs of caries activity increases from about 20-28% when adopting the 2013 criteria compared to the 2003 criteria. However, the associations between ethnicity, SEP and tooth decay did not change when using the 2003 criteria (See Table S6) compared to the associations reported in Table 2 (which use the 2013 criteria).

*4. Gingivitis and Plaque:* The pattern of tooth decay by ethnicity and SEP was also reflected by the analysis of plaque (Table S8). Among children aged 5, the predicted probability of plaque was higher in Pakistani and Bangladeshi children. There were no statistically significant socioeconomic differences in levels of plaque among children aged 5, 8 and 12. However, among children aged 15, predicted probability of plaque increased with greater area deprivation. Residential deprivation was also associated with gingivitis at age 12 and 15 (Table S7). Black African children aged 8 and 12 had the lowest predicted probability of gingivitis, but there was no evidence of ethnic differences among children aged 15.

*5. Multilevel analyses:* In order to analyse the relatively smaller numbers of cases in some of the ethnic groups, we also conducted multilevel logistic analyses as a sensitivity check, analyzing the presence of dental decay (using the 2013 criteria) at the tooth level (level 1) clustered within children (level 2). In Table S10, we show the results for the analysis of children aged 5 (analyzing whether they had decay in their primary teeth) and of children aged 15 (analyzing whether they had decay in their permanent teeth). At age 5, similar to the analysis shown in Table 2, a number of ethnic minority children had significantly higher odds of decay in their primary teeth. Socioeconomically disadvantaged children aged 5 also had higher odds of decay. However, among children aged 15, there were no ethnic differences in decayed teeth. The association between dental decay and free school meals eligibility was smaller among 15-year-old children compared to 5-year-old children, but the association with deprived school and IMD rank is very similar at both ages.

**Table S5.** Survey weighted percentages of children with clinical decay (including visual and cavitated enamel) criteria 2013; and obvious decay (visual and cavitated dentine) criteria 2003 (CDHS 2013)

| **Age** | **2003 criteria** | **2013 criteria** | **Increase in decay comparing 2013 criteria with 2003 criteria** |
| --- | --- | --- | --- |
| 5-year-olds | 27.9% | 46.9% | 19.0% |
| 8-year-olds | 41.8% | 63.0% | 21.1% |
| 12-year-olds | 23.3% | 51.1% | 27.8% |
| 15-year-olds | 22.0% | 50.4% | 28.2% |
| Overall | 28.7% | 52.7% | 24.0% |

**Table S6.** Predicted Rates (PRs) and 95% Confidence Intervals (CIs) from negative binomial regression models^a,b^: obvious tooth decay (2003 criteria) in children regressed on ethnicity/SEP in the four age-cohort samples (CDHS 2013)

|  | **N=2,217** | **N=2,083** | **N=2,183** | **N=2,058** | **Age interaction** |
| --- | --- | --- | --- | --- | --- |
| **Variables** | **5yrs** | **8yrs** | **12yrs** | **15yrs** | **ethnicity/SEP** |
|  | **PR (95% CI)** | **PR (95% CI)** | **PR (95% CI)** | **PR (95% CI)** |  |
| **Ethnicity** |  |  |  |  |  |
| White British & Irish | 0.78 (0.65, 0.91) | 1.20 (1.05, 1.34) | 0.63 (0.43, 0.83) | 0.56 (0.39, 0.73) |  |
| Other White | 0.98 (0.56, 1.39) | 1.85 (0.70, 2.99) | 1.04 (0.42, 1.67) | 0.30 (0.13, 0.46) |  |
| Mixed White | 0.94 (0.48, 1.39) | 0.92 (0.57, 1.27) | 0.41 (0.14, 0.69) | 0.65 (0.28, 1.03) |  |
| Indian | 1.46 (0.38, 2.54) | 1.76 (0.40, 3.13) | **0.20 (0.09, 0.31)**** | **0.16 (0.08, 0.24)***** | |
| Pakistani | **1.95 (1.01, 2.95)***** | **1.79 (1.28, 2.30)*** | 0.48 (0.18, 0.78) | **0.29 (0.12, 0.46)*** |  |
| Bangladeshi | 1.37 (0.06, 2.69) | 1.56 (0.58, 2.54) | 0.32 (0.01, 0.63) | 0.39 (0.09, 0.69) |  |
| Black African | **0.19 (0.05, 0.34)***** | 0.67 (0.15, 1.18) | **0.16 (0.02, 0.29)**** | 0.44 (-0.04, 0.91) |  |
| Black Caribbean | **0.30 (0.12, 0.47)**** | **0.38 (0.08, 0.68)**** | **0.20 (0.06, 0.34)**** | 0.35 (-0.17, 0.88) |  |
| *F test p value (df)* | ***<0.001 (7)*** | ***<0.001 (7)*** | ***<0.001 (7)*** | ***<0.001 (7)*** | ***<0.001 (21)*** |
| **Free school meal** |  |  |  |  |  |
| not eligible | 0.67 (0.57, 0.77) | 1.17 (1.03, 1.30) | 0.43 (0.34, 0.53) | 0.47 (0.34, 0.59) |  |
| eligible | **1.27 (0.93, 1.61)***** | 1.43 (1.14, 1.72) | **1.01 (0.68, 1.34)***** | **0.65 (0.44, 0.86)*** |  |
| *F test p value (df)* | ***<0.001 (1)*** | *0.052 (1)* | ***<0.001 (1)*** | ***0.04 (1)*** | ***<0.001 (3)*** |
| **Deprived school** |  |  |  |  |  |
| no | 0.70 (0.56, 0.83) | 1.20 (1.04, 1.37) | 0.57 (0.40, 0.74) | 0.41 (0.29, 0.52) |  |
| yes | **1.05 (0.76, 1.35)*** | 1.26 (1.03, 1.49) | 0.58 (0.34, 0.83) | **0.82 (0.44, 1.20)*** |  |
| *F test p value (df)* | *0.03 (1)* | *0.69 (1)* | *0.92 (1)* | ***0.007 (1)*** | ***0.004 (3)*** |
| **IMD rank** |  |  |  |  |  |
| Least deprived | 0.43 (0.28, 0.57) | 0.85 (0.61, 1.09) | 0.31 (0.21, 0.40) | 0.34 (0.10, 0.57) |  |
| Most deprived | **1.54 (1.04, 2.03)***** | **1.92 (1.42, 2.42)**** | **1.06 (0.53, 1.60)***** | 0.77 (0.48, 1.06) |  |
| *F test p value (df)* | ***<0.001 (1)*** | ***0.002 (1)*** | ***<0.001 (1)*** | *0.11 (1)* | *0.52 (3)* |

^a^ Survey weighted models include ethnicity, all socioeconomic variables, sex, country, urban/rural, and number of permanent and primary teeth

^b^ Negative binomial regression models include an offset (log number of teeth).

* *p*< .05; ** *p*< .01; *** *p*< .001.

**Table S7.** Predicted Probabilities (PPs) and 95% Confidence Intervals (CIs) from probit regression models^a^: gingivitis in children regressed on ethnicity/SEP in the four age-cohort samples (CDHS 2013)

|  | **N=2,217** | **N=2,083** | **N=2,183** | **N=2,058** | **Age interaction** |
| --- | --- | --- | --- | --- | --- |
| **Variables** | **5yrs** | **8yrs** | **12yrs** | **15yrs** | **ethnicity/SEP** |
|  | **PR (95% CI)** | **PR (95% CI)** | **PR (95% CI)** | **PR (95% CI)** |  |
| **Ethnicity** |  |  |  |  |  |
| White British & Irish | 0.24 (0.17, 0.32) | 0.51 (0.43, 0.58) | 0.63 (0.54, 0.72) | 0.56 (0.46, 0.65) |  |
| Other White | 0.22 (0.11, 0.34) | 0.52 (0.38, 0.65) | 0.71 (0.57, 0.85) | 0.44 (0.30, 0.57) |  |
| Mixed White | 0.25 (0.15, 0.35) | 0.40 (0.24, 0.55) | 0.60 (0.39, 0.81) | 0.41 (0.15, 0.66) |  |
| Indian | 0.28 (0.09, 0.46) | 0.60 (0.35, 0.85) | 0.54 (0.35, 0.72) | 0.53 (0.30, 0.76) |  |
| Pakistani | 0.22 (0.09, 0.35) | 0.48 (0.32, 0.63) | 0.61 (0.38, 0.83) | 0.63 (0.42, 0.84) |  |
| Bangladeshi | 0.38 (0.14, 0.62) | 0.39 (0.15, 0.63) | 0.48 (0.17, 0.78) | **0.41 (0.25, 0.56)*** |  |
| Black African | **0.07 (-0.02, 0.16)*** | **0.08 (0.01, 0.17)***** | **0.41 (0.21, 0.60)*** | 0.70 (0.43, 0.97) |  |
| Black Caribbean | **0.10 (0.03, 0.18)*** | **0.21 (0.01, 0.41)*** | 0.57 (0.32, 0.83) | 0.48 (0.28, 0.68) |  |
| *F test p value, (df)* | ***0.04 (7)*** | ***<0.001 (7)*** | ***0.03 (7)*** | *0.18 (7)* | *0.12 (21)* |
| **Free school meal** |  |  |  |  |  |
| not eligible | 0.22 (0.16, 0.29) | 0.48 (0.42, 0.54) | 0.61 (0.52, 0.71) | 0.54 (0.45, 0.63) |  |
| eligible | 0.28 (0.17, 0.39) | 0.46 (0.34, 0.58) | 0.62 (0.52, 0.71) | 0.57 (0.43, 0.71) |  |
| *F test p value, (df)* | *0.23 (1)* | *0.72 (1)* | *0.91 (1)* | *0.59 (1)* | *0.68 (3)* |
| **Deprived school** |  |  |  |  |  |
| no | 0.23 (0.17, 0.30) | 0.47 (0.39, 0.54) | 0.61 (0.05, 0.73) | 0.54 (0.43, 0.65) |  |
| yes | 0.24 (0.15, 0.32) | 0.53 (0.46, 0.60) | 0.62 (0.53, 0.72) | 0.57 (0.42, 0.72) |  |
| *F test p value,( df)* | *0.97 (1)* | *0.14 (1)* | *0.89 (1)* | *0.79 (1)* | *0.79 (3)* |
| **IMD rank** |  |  |  |  |  |
| Least deprived | 0.18 (0.09, 0.30) | 0.47 (0.37, 0.57) | 0.52 (0.42, 0.62) | 0.39 (0.25, 0.52) |  |
| Most deprived | 0.33 (0.20, 0.46) | 0.50 (0.38, 0.62) | **0.73 (0.60, 0.86)*** | **0.75 (0.65, 0.85)***** | |
| *F test p value, (df)* | *0.06 (1)* | *0.70 (1)* | ***0.02 (1)*** | ***0.003 (1)*** | *0.09 (3)* |

^a^ Survey weighted models include ethnicity, all socioeconomic variables, sex, country, urban/rural, and number of permanent and primary teeth

* *p*< .05; ** *p*< .01; *** *p*< .001.

**Table S8.** Predicted Probabilities (PPs) and 95% Confidence Intervals (CIs) from probit regression models^a^: plaque in children regressed on ethnicity/SEP in the four age-cohort samples (CDHS 2013)

|  | **N=2,217** | **N=2,083** | **N=2,183** | **N=2,058** | **Age interaction** |
| --- | --- | --- | --- | --- | --- |
| **Variables** | **5yrs** | **8yrs** | **12yrs** | **15yrs** | **ethnicity/SEP** |
|  | **PR (95% CI)** | **PR (95% CI)** | **PR (95% CI)** | **PR (95% CI)** |  |
| **Ethnicity** |  |  |  |  |  |
| White British & Irish | 0.33 (0.25, 0.40) | 0.56 (0.49, 0.63) | 0.54 (0.46, 0.62) | 0.41 (0.34, 0.48) |  |
| Other White | 0.24 (0.11, 0.37) | 0.63 (0.51, 0.76) | 0.59 (0.38, 0.80) | 0.30 (0.16, 0.43) |  |
| Mixed White | 0.45 (0.30, 0.61) | 0.68 (0.49, 0.87) | 0.54 (0.37, 0.70) | 0.32 (0.16, 0.47) |  |
| Indian | 0.32 (0.11, 0.52) | 0.57 (0.26, 0.88) | 0.46 (0.30, 0.61) | 0.31 (0.07, 0.54) |  |
| Pakistani | 0.50 (0.27, 0.72) | 0.63 (0.45, 0.80) | 0.43 (0.18, 0.68) | 0.32 (0.08, 0.55) |  |
| Bangladeshi | 0.54 (0.28, 0.81) | 0.60 (0.29, 0.91) | 0.63 (0.45, 0.81) | 0.33 (0.23, 0.42) |  |
| Black African | 0.21 (0.04, 0.39) | 0.49 (0.24, 0.74) | 0.40 (0.18, 0.61) | 0.39 (0.21, 0.57) |  |
| Black Caribbean | 0.24 (0.11, 0.38) | 0.51 (0.25, 0.77) | 0.67 (0.48, 0.85) | 0.22 (-0.06, 0.50) |  |
| *F test p value (df)* | ***0.009 (7)*** | *0.44 (7)* | *0.61 (7)* | *0.31 (7)* | ***<0.001 (21)*** |
| **Free school meal** |  |  |  |  |  |
| not eligible | 0.32 (0.25, 0.39) | 0.57 (0.51, 0.63) | 0.53 (0.45, 0.61) | 0.38 (0.30, 0.45) |  |
| eligible | 0.36 (0.24, 0.47) | 0.57 (0.45, 0.68) | 0.56 (0.48, 0.64) | 0.44 (0.35, 0.52) |  |
| *F test p value (df)* | *0.41 (1)* | *0.93 (1)* | *0.21 (1)* | *0.27 (1)* | *0.86 (3)* |
| **Deprived school** |  |  |  |  |  |
| no | 0.32 (0.23, 0.40) | 0.56 (0.49, 0.63) | 0.53 (0.43, 0.62) | 0.38 (0.30, 0.45) |  |
| yes | 0.37 (0.26, 0.47) | 0.61 (0.52, 0.70) | 0.57 (0.46, 0.67) | 0.42 (0.27, 0.57) |  |
| *F test p value (df)* | *0.45 (1)* | *0.33 (1)* | *0.58 (1)* | *0.63 (1)* | *0.99 (3)* |
| **IMD rank** |  |  |  |  |  |
| Least deprived | 0.34 (0.22, 0.46) | 0.62 (0.51, 0.72) | 0.49 (0.37, 0.62) | 0.27 (0.18, 0.36) |  |
| Most deprived | 0.31 (0.19, 0.42) | 0.50 (0.38, 0.61) | 0.59 (0.43, 0.75) | **0.55 (0.41, 0.69)**** | |
| *F test p value (df)* | *0.75 (1)* | *0.2 (1)* | *0.45 (1)* | ***0.007 (1)*** | *0.07 (3)* |

^a^ Survey weighted models include ethnicity, all socioeconomic variables, sex, country, urban/rural, and number of permanent and primary teeth

* *p*< .05; ** *p*< .01.

**Table S9.** Relative Index of Inequality (RII, from ranked Index of Multiple Deprivation) for tooth decay, filled teeth, plaque, gingivitis and poor periodontal health, by country, with p-values for the interaction term between RII and country (CDHS 2013)

|  |  | **age 5** | **age 8** | **age 12** | **age 15** |
| --- | --- | --- | --- | --- | --- |
| **Tooth decay** | England | 2.31 | 2.02 | 3.06 | 1.75 |
|  | Wales | 1.78 | 0.88 | 2.52 | 1.55 |
|  | Northern Ireland | 2.49 | 1.03 | 0.91 | 1.16 |
|  | *p-value country*RII (2df)* | *0.70* | *0.06* | *0.02* | *0.78* |
|  |  |  |  |  |  |
| **Filled teeth** | England | 2.46 | 1.30 | 1.08 | 2.59 |
|  | Wales | 2.60 | 1.03 | 1.32 | 1.42 |
|  | Northern Ireland | 5.10 | 0.77 | 2.11 | 1.77 |
|  | *p-value country*RII (2df)* | *0.68* | *0.67* | *0.29* | *0.46* |
|  |  |  |  |  |  |
| **Gingivitis** | England | 1.84 | 1.15 | 1.95 | 2.87 |
|  | Wales | 0.60 | 0.68 | 0.85 | 1.74 |
|  | Northern Ireland | 0.70 | 0.87 | 1.17 | 1.35 |
|  | *p-value country*RII (2df)* | *0.08* | *0.64* | *0.17* | *0.32* |
|  |  |  |  |  |  |
| **Plaque** | England | 0.93 | 0.72 | 1.30 | 2.20 |
|  | Wales | 0.62 | 0.75 | 1.63 | 2.00 |
|  | Northern Ireland | 1.02 | 0.74 | 0.69 | 0.97 |
|  | *p-value country*RII (2df)* | *0.77* | *0.99* | *0.11* | *0.34* |
|  |  |  |  |  |  |
| **Poor perio** | England | 1.01 | 0.81 | 1.81 | 2.59 |
|  | Wales | 0.71 | 0.89 | 0.97 | 2.01 |
|  | Northern Ireland | 0.91 | 0.66 | 0.68 | 1.43 |
|  | *p-value country*RII (2df)* | *0.85* | *0.84* | *0.03* | *0.63* |

**Table S10**. Odds ratios (ORs) and 95% Confidence Intervals (CIs) of decay in primary teeth of children aged 5 and permanent teeth of children aged 15, by ethnicity and socioeconomic position (CDHS 2013): Multilevel model^a^ of teeth (level 1) clustered within children (level 2)

| **Explanatory variables** | **age 5** | **age 15** |
| --- | --- | --- |
|  | Obs N=43,274  Groups N=2,313 | Obs N=58,926  Groups N=2,113 |
| **Ethnicity (ref: White British & Irish)** | |  |
| Gypsy or Irish Travellers | 2.39 (0.72, 7.86) | 5.45 (0.89, 33.48) |
| Other White background | **2.01 (1.34, 3.03)**** | 0.98 (0.57, 1.69) |
| White and Black Caribbean | 0.44 (0.19, 1.02) | 0.52 (0.17, 1.61) |
| White and Black African | 0.77 (0.26, 2.26) | 1.71 (0.54, 5.37) |
| White and Asian | **2.39 (1.05, 5.44)*** | 0.95 (0.36, 2.51) |
| Other Mixed background | 1.17 (0.42, 3.31) | 0.66 (0.28, 1.56) |
| Indian | **2.62 (1.22, 5.63)*** | 0.57 (0.32, 1.02) |
| Pakistani | **3.19 (1.88, 5.40)***** | 1.06 (0.68, 1.64) |
| Bangladeshi | 1.36 (0.64, 2.88) | 0.78 (0.47, 1.29) |
| Chinese | 1.72 (0.5, 5.94) | 1.56 (0.05, 45.43) |
| Other Asian | **5.94 (3.01, 11.73)***** | 1.00 (0.35, 2.86) |
| Black African | **0.39 (0.18, 0.83)*** | 1.02 (0.56, 1.85) |
| Black Caribbean | 0.27 (0.07, 1.04) | 0.45 (0.18, 1.1) |
| Other Black/African/Caribbean | 0.75 (0.35, 1.63) | 0.66 (0.29, 1.51) |
| Arab | 3.64 (0.82, 16.27) | 1.01 (0.24, 4.22) |
| Unspecified other ethnic groups | **4.67 (2.01, 10.90)***** | 1.19 (0.58, 2.46) |
| **Free School Meal Eligibility (ref: no)** | |  |
| yes | **1.74 (1.40, 2.15)***** | **1.30 (1.06, 1.58)*** |
| **Deprived school (ref: no)** |  |  |
| yes | **1.36 (1.10, 1.69)**** | **1.43 (1.16, 1.75)**** |
| **RII_IMD (ref: RII=0)** |  |  |
| RII=1 | **1.69 (1.16, 2.46)**** | **1.76 (1.23, 2.52)**** |

^a^ Models include ethnicity, all socioeconomic variables, sex, country, urban/rural, and number of permanent and primary teeth

* *p*< .05; ** *p*< .01; *** *p*< .001.

**Figure S1.** Predicted Rates and 95% Confidence Intervals of tooth decay by White British/Irish, Other White, Indian and Pakistani ethnic groups: children aged 5 and 15 (from Table 2) (CDHS 2013)

**References**

1. Mathur R, Grundy E, Smeeth L. Availability and use of uk based ethnicity data for health research. 2013. NCRM Working Paper. n/a. (Unpublished). <http://eprints.ncrm.ac.uk/3040/>. Accessed 20 September 2017.

2. Office for National Statistics. Ethnicity and national identity in England and Wales: 2011. 2012. https://www.ons.gov.uk/peoplepopulationandcommunity/culturalidentity/ethnicity/article/ethnicityandnationalidentityinenglandandwales/2012-12-11. Accessed 20 September 2017.

3. Office for National Statistics. 2011 census analysis: Ethnicity and religion of the non-uk born population in England and Wales. 2015. http://webarchive.nationalarchives.gov.uk/20160105160709/http://www.ons.gov.uk/o ns/dcp171776_407038.pdf. Accessed 20 September 2017.

4. Office for National Statistics. Area classifications, 2011. 2011. <http://webarchive.nationalarchives.gov.uk/20160105160709/http://www.ons.gov.uk/ons/guide-method/geography/products/area-classifications/ns-area-classifications/ns-2011-area-classifications/index.html>. Accessed 20 September 2017.

5. Finney N, Simpson L. "Sleepwalking to segregation’? Challenging myths about race and migration. Bristol: *Policy Press*. 2009; 224.

6. Department for Communities and Local Government. English indices of deprivation 2010. 2011. https://www.gov.uk/government/statistics/english-indices-of-deprivation-2010. Accessed 20 September 2017.

7. Welch Government. Welsh index of multiple deprivation 2011. 2011. https://statswales.gov.wales/Catalogue/Community-Safety-and-Social-Inclusion/Welsh-Index-of-Multiple-Deprivation/Archive/WIMD-2011. Accessed 20 September 2017.

8. Northern Ireland Statistics and Research Agency. Northern Ireland multiple deprivation measure 2010. 2010. https://www.nisra.gov.uk/statistics/deprivation/northern-ireland-multiple-deprivation-measure-2010-nimdm2010. Accessed 20 September 2017.
